# 

**Published:** 1869-02

**Authors:** 


### CONTENTS.

ORIGINAL COMMUNICATIONS.

Address at the Commencement of the Ohio College of Dental Surgery .......... 53
Suppuration .......... 58
Chloro-Nitrous Oxyd .......... 60
Imperfect Enamel .......... 62

CORRESPONDENCE.

"Southern Dental Association" .......... 64

PROCEEDINGS OF SOCIETIES.

Ohio State Dental Association .......... 67
New York State Dental Society .......... 75

SELECTIONS.

The Principles Regulating the Natural Evacuation of Abscesses .......... 77
The New Webster .......... 80
Hypodermic Medication .......... 81
New Modes of Preparing Objects for the Microscope .......... 84
Flexible Sulphur .......... 84
The Therapeutics of Wakefulness .......... 85
A New Alloy of Aluminum .......... 88
Sore Gums or Throat .......... 88
Depression and Replacement of the Superior Maxilla (Langenbeck's Operation) .......... 89

EDITORIAL.

The Present Status of the Rubber Question .......... 91
Calcification of Tooth Pulp .......... 92
The Rubber Dam .......... 93
Meeting of the Chicago Microscopical Club—Structure of the Blood Corpuscles .......... 94
Meeting of Dentists—Resolutions of Respect .......... 96
Obituary .......... 96

